# P-1511. Pediatric inpatient influenza vaccination before and during the COVID-19 pandemic

**DOI:** 10.1093/ofid/ofaf695.1695

**Published:** 2026-01-11

**Authors:** Suchitra Rao, Ravi Jhaveri, Laura Pyle, Christina R Studts, Ashleigh Lewis, Bryan Strub, Alexa Mendoza, Marisa Payan, Annika Hofstetter

**Affiliations:** University of Colorado School of Medicine, Aurora, CO; Ann & Robert H. Lurie Children's Hospital of Chicago, Chicago, IL; University of Washington, Seattle, Washington; University of Colorado School of Medicine, Aurora, CO, Aurora, Colorado; Seattle Children's Hospital, Seattle, Washington; Seattle Children's Research Institute, Seattle, Seattle, Washington; Lurie Children's Hospital, Chicago, Illinois; Children's Hospital Colorado, Aurora, Colorado; Seattle Children's Hospital, Seattle, Washington

## Abstract

**Background:**

Annual influenza vaccination is recommended for all individuals >6 months of age during any healthcare seeking opportunity, including hospitalization. While influenza vaccination coverage has declined nationally since the COVID-19 pandemic, the impact of the pandemic on pediatric inpatient influenza vaccination is unknown. Our objective was to assess pediatric influenza vaccine screening and administration during hospitalization before and during/after the COVID-19 pandemic.

**Table 1.**
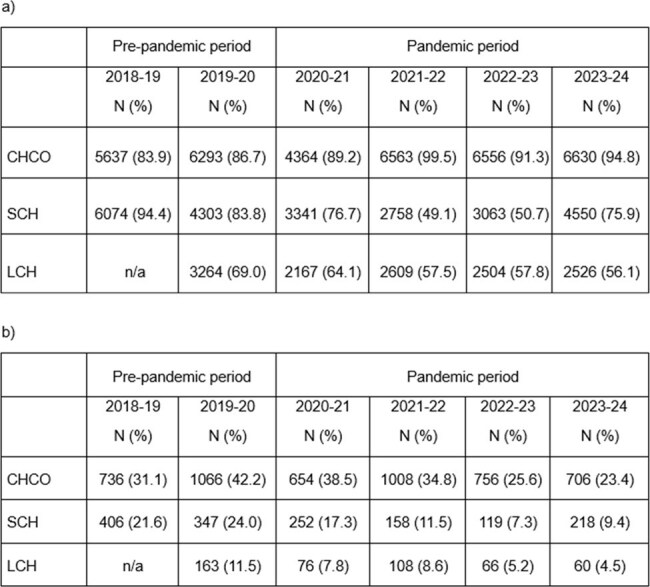
a) Influenza vaccination screening (among hospitalizations) and b) influenza vaccine administrations (among eligible inpatient visits) during the 2018-19 to 2023-24 seasons at Children’s Hospital Colorado, Seattle Children’s Hospital and Lurie Children’s Hospital CHCO- Children’s Hospital Colorado; SCH- Seattle Children’s Hospital; LCH- Lurie Children’s Hospital

**Figure:**
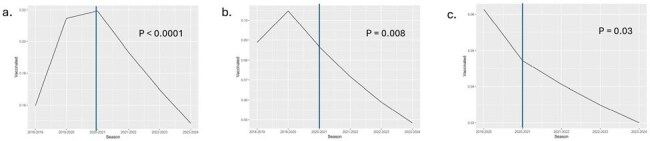
Breakpoint regression model for inpatient influenza vaccination receipt at a) Children’s Hospital Colorado, b) Seattle Children’s Hospital and c) Lurie Children’s Hospital using the 2020-21 season as the change point (blue line). Plots represent the estimated probability of influenza vaccine receipt over time. P values < 0.05 indicate a significant change in the outcome probabilities over time.

**Methods:**

We conducted a retrospective study of children aged 6 months-17 years admitted to a non-intensive care unit at Children’s Hospital Colorado, Seattle Children’s Hospital, or Lurie Children’s Hospital during the 2018-19 through 2023-24 seasons. We compared inpatient influenza vaccine screening and administration between the pre-pandemic (2018-2020) and pandemic (2020-2024) periods. We also assessed the change in rates over time using segmented modelling via breakpoint regression.

**Results:**

During the study period, 70,306 children were admitted to the three hospitals representing 94,719 hospitalizations. The median age of individuals was 7.1 years (IQR 2.4-13.4), 52.2% were male, 56.6% were White, 10.4% were Black, 5.4% were Asian, 35% were Hispanic. Most children were admitted to a medical unit (56.5%), followed by a surgical unit (19.7%). Overall, 25,571 (84.4%) were screened for influenza vaccination status during the pre-pandemic period, which declined during the pandemic period to 47,631 (73.9%). Among vaccine-eligible patients who were screened, 2,718 (28.2%) received a dose during the pre-pandemic period, which declined to 4,181(18.8%) during the pandemic period. Influenza vaccine administration was highest during the 2019-20 season. These screening and administration rates differed between the pre-pandemic and pandemic periods (Table 1). Regression modeling with segmented relationships demonstrated a decline in vaccine receipt across all three sites (Figure 1).

**Conclusion:**

There was a decline in influenza screening and vaccine administrations during the COVID-19 pandemic. These trends mirror outpatient findings and require tailored strategies to promote vaccination.

**Disclosures:**

Ravi Jhaveri, MD, AstraZeneca: Advisor/Consultant|Gilead: Advisor/Consultant|GSK: Grant/Research Support|PIDS: editorial stipend-JPIDS|Sanofi: Advisor/Consultant|Seqirus: Advisor/Consultant|UptoDate: royalties

